# Antibiotic Prescription for Acute Diverticulitis: Evaluation of Compliance With National Guidelines

**DOI:** 10.7759/cureus.92378

**Published:** 2025-09-15

**Authors:** Usman Mateen, Hassan Imtiaz, Maaz A Yusufi, Najaf Siddiqi, Paul Froggatt

**Affiliations:** 1 General Surgery, University Hospitals Dorset NHS Foundation Trust, Poole, GBR; 2 Trauma and Orthopaedics, University Hospitals Dorset NHS Foundation Trust, Poole, GBR; 3 Surgery, University Hospitals Dorset NHS Foundation Trust, Poole, GBR; 4 Colorectal Surgery, Poole Hospital, Poole, GBR

**Keywords:** antibiotic stewardship (ams), clinical audit, complicated diverticulitis, quality improvement (qi), uncomplicated diverticulitis

## Abstract

Introduction: Diverticulitis is a common surgical problem that can be categorised as uncomplicated or complicated diverticulitis. National guidelines for managing acute diverticulitis recommend that antibiotics should only be given to patients who are systemically unwell, have a background of immunosuppression or have complicated diverticulitis. We analysed the compliance with these guidelines at our hospital, identified gaps, and introduced appropriate measures to improve service provision.

Methods: This was a closed-loop audit. The first cycle was a retrospective analysis conducted between July and December 2024. Following the identification of gaps in practice, appropriate educational interventions were undertaken, and a second audit cycle was conducted to evaluate their impact. The second cycle was prospectively undertaken between February and May 2025. Adult patients with a computed tomography-confirmed diagnosis of acute diverticulitis met the inclusion criteria. Measured outcomes included demographics, signs and symptoms at presentation, relevant imaging, observations and antibiotic prescription. Standards were derived from National Institute for Health and Care Excellence (NICE) guidelines with target compliance set at 100%.

Results: Forty-three patients were evaluated during the first audit cycle. 24/43 (56%) patients had uncomplicated diverticulitis. Of these, 3/24 were immunocompromised and were appropriately prescribed antibiotics; all the remaining 21/24 patients with a competent immune system and systemically well were given antibiotics against the recommendations, resulting in non-compliance with standards. 19/43 (44%) had complicated diverticulitis, with all receiving antibiotics as recommended, therefore resulting in 100% compliance. Following appropriate interventions, the subsequent audit cycle was conducted, where 35 patients were included in the second cycle. 26/35 had uncomplicated diverticulitis; of these, 8 (31%) required antibiotics due to systemic illness or immunosuppression, 12 (46%) were not prescribed antibiotics in line with the standard, while 6 (23%) unnecessarily received antibiotics against the standard. Compliance therefore improved from 0% to 66% between audit cycles with regards to managing uncomplicated diverticulitis. All nine of the remaining 35 patients with complicated diverticulitis received appropriate antibiotics, achieving 100% compliance, consistent with the previous audit cycle.

Conclusions: This quality improvement project highlights the importance of differentiating amongst complicated and uncomplicated diverticulitis, which therefore dictates management, and potentially avoids unnecessary administration of intravenous antibiotics and hospital stay. This article aims to increase awareness among relevant surgical teams in order to improve patient outcomes and reduce pressures on the health care system.

## Introduction

Acute diverticulitis is a common inflammatory condition of the colon that significantly impacts healthcare systems in Western countries [1]. It is estimated that approximately 10-25% of individuals with colonic diverticulosis will develop diverticulitis at some point in their lives, with the majority presenting to emergency departments with abdominal pain and systemic symptoms [2]. The condition is broadly categorised into uncomplicated diverticulitis, involving local inflammation only, and complicated diverticulitis, which may involve perforation, abscess, fistula, peritonitis, or obstruction [3].

Traditionally, all patients with acute diverticulitis were treated with bowel rest and empiric broad-spectrum antibiotics. However, recent high-quality randomised controlled trials and guideline updates have shifted this paradigm, particularly in the management of uncomplicated diverticulitis [4-6]. The National Institute for Health and Care Excellence (NICE), in its guidance NG147, recommends that patients with uncomplicated diverticulitis who are systemically well and immunocompetent should not receive antibiotics, as evidence suggests no significant benefit in recovery time or complication rates. Instead, antibiotics should be reserved for those who are systemically unwell, immunosuppressed, or have evidence of complicated disease [7].

Despite these clear recommendations, several studies have demonstrated a persistent overuse of antibiotics in uncomplicated diverticulitis, both in primary and secondary care settings [8-10]. This trend not only contradicts current best practices but also contributes to antimicrobial resistance, which is a growing global health threat [11]. Inappropriate antibiotic use is associated with adverse drug reactions, disruption of gut microbiota, increased incidence of *Clostridium difficile* infection, and unnecessary healthcare costs [12-14].

Addressing this practice gap requires a systematic approach through clinical audit, education, and feedback, which are fundamental tools in quality improvement methodology [15]. Closed-loop audits or quality improvement projects (QIPs), where initial data collection is followed by intervention and re-audit, are particularly effective in modifying clinical practice and aligning practice with national standards [16]. With this in mind, we conducted a quality improvement project (QIP) at our hospital to assess adherence to NICE guidelines on antibiotic prescribing in computed tomography (CT) confirmed cases of acute diverticulitis. We aimed to quantify baseline compliance, implement targeted interventions, and evaluate their impact in a subsequent audit cycle. By doing so, we sought to improve the quality of care, reduce unnecessary antibiotic exposure, and contribute to antimicrobial stewardship efforts in surgical practice.

## Materials and methods

This was a closed-loop quality improvement project conducted under the General Surgery department at Poole Hospital, University Hospitals Dorset NHS Foundation Trust, United Kingdom.

The first cycle was conducted retrospectively, between July and December 2024. Following identification of gaps in practice (detailed in Results section), appropriate educational interventions were undertaken. These included display of posters in relevant clinical areas, such as emergency department and general surgical wards. A dedicated teaching session conducted by a Senior Registrar was delivered to the General Surgical doctors, highlighting the importance of differentiating amongst complicated and uncomplicated diverticulitis, and following management principles in line with recommendations from NICE [7]. Following these, a second audit cycle was conducted to evaluate the impact of these interventions. This was prospectively undertaken between February and May 2025.

Adult patients (age >18 years) with a CT-confirmed diagnosis of acute diverticulitis met the inclusion criteria. Patients who had incomplete records, a lack of a CT scan-confirmed diagnosis, and those managed in outpatient settings were excluded.

Audit standards were derived from NICE guidelines NG147 [7], which are given in Table 1. Data sources included electronic patient records (EPRs) and electronic prescription and medicine administration records (EPMAs). Measured outcomes included patient demographics, CT findings, observations at presentation, immunocompetent status, and antibiotic prescription.

**Table 1:** Audit standards.

Audit standard	Target compliance (%)
Patients with acute uncomplicated diverticulitis who are systemically well and not immunocompromised should not receive antibiotics	100%
Patients with acute complicated diverticulitis or those with uncomplicated diverticulitis who are immunocompromised or "systemically unwell" should receive antibiotics	100%

Data was collected, stored and analysed using Microsoft Excel (Microsoft Corporation, Redmond, Washington, USA). Patient identifiers were removed from the datasheet prior to final analysis. Chi-squared test was employed to statistically compare the results of the first and second loop results, with a p-value of less than 0.05 being set as the mark for statistical significance.

## Results

A total of 43 patients were evaluated during the first audit cycle (July to December 2024). The mean age was 65 years, with a symmetrical gender distribution of 22 (51%) females and 21 (49%) males. Forty-three patients were evaluated during the first audit cycle. 24/43 (56%) patients had uncomplicated diverticulitis. Of these, 3/24 were immunocompromised and were appropriately prescribed antibiotics; all the remaining 21/24 patients with a competent immune system and systemically well were given antibiotics against the recommendations, resulting in non-compliance with audit standards. 19/43 (44%) had complicated diverticulitis, with all receiving antibiotics as recommended, therefore resulting in 100% compliance.

Following appropriate interventions, the subsequent audit cycle was conducted, where 35 patients were included in the second cycle. Mean age was 66 years, with 22/35 (63%) females. 26/35 had uncomplicated diverticulitis; of these, 8 (31%) required antibiotics due to systemic illness or immunosuppression, 12 (46%) were not given antibiotics as per standard, while 6 (23%) received antibiotics against the standard. Compliance therefore improved from 0% to 77% between audit cycles with regards to managing uncomplicated diverticulitis. All remaining 9/35 patients with complicated diverticulitis appropriately received antibiotics, conferring 100% compliance, similar to the previous audit cycle.

In the second cycle (February to May 2025), 35 patients were included. Of the 26 patients with uncomplicated diverticulitis, eight had valid indications for antibiotics due to systemic illness or immunosuppression and were excluded from compliance analysis. Among the remaining 18, 12 patients did not receive antibiotics (compliant), and six received antibiotics without indication (non-compliant), leading to a 66% compliance rate for this subgroup. The remaining nine patients with complicated diverticulitis all received appropriate antibiotics, maintaining 100% compliance. Patient demographics between the two audit cycles are presented in Table 2.

**Table 2:** Patient demographics.

	First audit cycle (N = 43)	Second audit cycle (N = 35)
Age (Mean ± SD)	65 ± 14.9	66 ± 16.2
Gender		
Female (n)	22	22
Male (n)	21	13
Uncomplicated diverticulitis and immunocompetent/not systemically unwell (n)	21	18
Antibiotics not given	0	12
Antibiotics given	21	6
Uncomplicated diverticulitis and immunocompromised/systemically unwell (n)	3	8
Antibiotics not given	0	0
Antibiotics given	3	8
Complicated diverticulitis (n)	19	9

The compliance against audit standards between the two cycles is presented in Table 3.

**Table 3:** Compliance against audit standards.

Audit standard(s)	Observed compliance (%)		Target compliance (%)
	First audit cycle (n, %)	Second audit cycle (n, %)	
Patients with acute uncomplicated diverticulitis who are systemically well and not immunocompromised, should not receive antibiotics	0/21 (0%)	12/18 (66%)	100%
Patients with acute complicated diverticulitis or those with uncomplicated diverticulitis who are immunocompromised or 'systemically unwell' should receive antibiotics	22/22 (100%)	17/17 (100%)	100%

A Chi-square test was employed to compare compliance for each audit standard between the two audit cycles. The improvement in correctly identifying uncomplicated diverticulitis patients and not prescribing them antibiotics as per recommendations was a statistically significant change (p = 0.0001). Given that all patients with complicated diverticulitis or those with uncomplicated diverticulitis but having either an immunocompromised state or being systemically unwell, were given antibiotics appropriately between both audit cycles, with optimal compliance at 100% each, no change was observed, therefore yielding a p-value of 1.0. These results are given in Table 4.

**Table 4:** Statistical analysis. *Chi-squared test used for assessing statistical significance. **p-value of <0.05 set as measure of statistical significance.

Audit standard(s)	p-value*	Statistically significant** (Yes/no)
Patients with acute uncomplicated diverticulitis who are systemically well and not immunocompromised should not receive antibiotics	0.0001	Yes
Patients with acute complicated diverticulitis or those with uncomplicated diverticulitis who are immunocompromised or systemically should receive antibiotics	1.0	No

## Discussion

Our audit aligns with the evolving paradigm in diverticulitis management: tailoring antibiotic use based on disease severity. Uncomplicated acute diverticulitis can often be managed conservatively without antibiotics, particularly in immunocompetent patients. A systematic review and meta-analysis showed that withholding antibiotics in such cases did not worsen outcomes; there were no significant differences in complication rates or need for interventions, although antibiotic use did prolong hospital stay [17]. Complementing this, recent European guidelines strongly recommend withholding antibiotics in uncomplicated diverticulitis, with structured advice on self-monitoring and clear instructions if symptoms recur [18]. Conversely, in complicated diverticulitis, antibiotic therapy, often with broad-spectrum intravenous antibiotics, is the standard of care, with CT imaging guiding escalation and duration [7].

Our audit highlights a potential gap in clinical practice, which may represent an overlooked domain in general surgery. An audit conducted in a Swiss tertiary hospital found guideline adherence in diverticulitis cases was strikingly low (11% locally, 18% internationally) [19]. These findings resonate with the findings in our project, highlighting the importance of ensuring that appropriate antibiotic prescribing is consistently applied in diverticulitis cases based on whether they are complicated or uncomplicated.

Clinical audit, similar to our study, possesses the potential to reshape antimicrobial prescribing. Nationally, a Delphi consensus-derived audit tool was developed to systematically assess antibiotic prescribing appropriateness in NHS acute hospitals. It effectively captures critical decision points: initial prescription, pre-72-hour review, and total course length. This provides an objective framework for identifying areas of overprescribing [20].

Through this article, we aim to increase awareness regarding identification of unwarranted antibiotic use, reinforcing national guideline adherence, and highlight targets for educational and system-level interventions. Ultimately, they support antimicrobial stewardship objectives: rationalising antibiotic use, minimising resistance development, enhancing patient safety, and reducing resource waste [21].

We strongly believe that our quality improvement project has the potential to influence clinical practice in general surgical departments with regards to compliance with guidelines for management of acute diverticulitis, with an added benefit of cost effectiveness given the potential reductions in overprescribing of intravenous antibiotics, thus resulting in fewer hospital admissions.

This project has several limitations. First, it was conducted as a single-centre audit with a modest sample size, which may limit generalisability of findings to other hospitals or regions with different patient demographics and care pathways. Second, the classification of diverticulitis as complicated versus uncomplicated, though guided by CT imaging, can involve some degree of subjectivity, potentially introducing variability into compliance assessment. Third, as with many quality improvement initiatives, the observed improvement may partly reflect a short-term effect of targeted education and increased awareness, and sustained gains will require ongoing reinforcement through regular audit. Finally, outcome measures were limited to prescribing compliance; longer-term endpoints such as hospital stay, recurrence rates, and adverse events were not assessed but would strengthen future studies.

## Conclusions

This closed-loop audit demonstrated that focused education and visibility of national guidelines significantly improved compliance with antibiotic prescribing standards for diverticulitis. Appropriate differentiation between complicated and uncomplicated cases is essential in guiding therapy, reducing unnecessary antibiotic exposure, and supporting antimicrobial stewardship. Our findings mirror international evidence that uncomplicated diverticulitis can be safely managed without antibiotics in immunocompetent, systemically well patients, while antibiotics remain vital for complicated disease or high-risk groups. Sustaining and scaling such initiatives will be critical not only for diverticulitis management but also for the wider agenda of reducing inappropriate antibiotic use and combating antimicrobial resistance across healthcare systems.
